# The Social Robot and the Digital Physiotherapist: Are We Ready for the Team Play?

**DOI:** 10.3390/healthcare9111454

**Published:** 2021-10-27

**Authors:** Rossella Simeoni, Federico Colonnelli, Veronica Eutizi, Matteo Marchetti, Elena Paolini, Valentina Papalini, Alessio Punturo, Alice Salvò, Nicoletta Scipinotti, Christian Serpente, Emanuele Barbini, Riccardo Troscia, Giovanni Maccioni, Daniele Giansanti

**Affiliations:** 1 Faculty of Medicine and Surgery, Università Cattolica del Sacro Cuore, San Martino al Cimino, 01100 Viterbo, Italy; rossella.simeoni.1955@gmail.com (R.S.); federico.colonnelli@hotmail.com (F.C.); veronica.eutizi.univ.catt.21@gmail.com (V.E.); matteo.marchetti.univ.catt.21@gmail.com (M.M.); elena.paolini.univ.catt.21@gmail.com (E.P.); valentina.papalini.univ.catt.21@hotmail.com (V.P.); alessio.punturo.univ.catt.21@hotmail.com (A.P.); alice.salvo.univ.catt.21@hotmail.com (A.S.); n.scipinotti.univ.catt@hotmail.com (N.S.); c.serpente.univ.catt@hotmail.com (C.S.); emanuele.barbini.univ.catt.21@hotmail.com (E.B.); r.troscia.univ.catt@hotmail.com (R.T.); 2 Centre Tisp, Istituto Superiore di Sanità, 00161 Rome, Italy; giovanni.maccioni@iss.it

**Keywords:** e-health, medical devices, m-health, rehabilitation, robotics, organization models, artificial intelligence, electronic surveys, social robots, collaborative robots

## Abstract

*Motivation*: We are witnessing two phenomena. The first is that the physiotherapist is increasingly becoming a figure that must interact with Digital Health. On the other hand, social robots through research are improving more and more in the aspects of social interaction thanks also to artificial intelligence and becoming useful in rehabilitation processes. It begins to become strategic to investigate the intersections between these two phenomena. *Objective*: Therefore, we set ourselves the goal of investigating the consensus and opinion of physiotherapists around the introduction of social robots in clinical practice both in rehabilitation and assistance. *Procedure*: An electronic survey has been developed focused on social robot-based rehabilitation and assistance and has been submitted to subjects focused on physiotherapy sciences to investigate their opinion and their level of consent regarding the use of the social robot in rehabilitation and assistance. Two samples of subjects were recruited: the first group (156 participating subjects, 79 males, 77 females, mean age 24.3 years) was in the training phase, and the second (167 participating subjects, 86 males, 81 females, mean age 42.4 years) group was involved in the work processes. An electronic feedback form was also submitted to investigate the acceptance of the proposed methodology. *Results*: The survey showed a consistency of the results between the two samples from which interesting considerations emerge. Contrary to stereotypes that report how AI-based devices put jobs at risk, physiotherapists are not afraid of these devices. The subjects involved in the study believe the following: (a) social robots can be reliable co-workers but will remain a complementary device; (b) their role will be of the utmost importance as an operational manager in their use and in performance monitoring; (c) these devices will allow an increase in working capacity and facilitate integration. All those involved in the study believe that the proposed electronic survey has proved to be a useful and effective tool that can be useful as a periodic monitoring tool and useful for scientific societies. *Conclusions*: The evolution of social robots represents an unstoppable process as does the increase in the aging of the population. Stakeholders must not look with suspicion toward these devices, which can represent an important resource, but rather invest in monitoring and consensus training initiatives.

## 1. Introduction

Robotics has made it possible to introduce social robots (SRs) in both remote rehabilitation and assistance as a valid support in several sectors both as a direct and practical support and as a mediator [1,2,3]. The SR also stands as one of the key tools in rehabilitation through robotics as highlighted in the special issue Rehabilitation and Robotics: Are They Working Well Together? [4], of which this study aims to be a part.

It is natural that with this evolution, it is important to reflect on new professional figures or at least on the remodeling of already existing professional figures. One of the key figures in physical rehabilitation and assistance is that of the physiotherapist, who stands between the physician of physical medicine and rehabilitation and the patient, entering with greater contact with the patient. New models of care emerged, during the COVID-19 pandemic, based on technologies that allow greater social distancing between the patient and the therapist. Based on this, an expansion of the job description of many figures involved in rehabilitation and assistance is emerging. This is closely related to the remodeling of the *work-flow* that SRs have the potential to modify. Changes in the *work-flow* have a direct impact on the job description of the worker and therefore on the tasks he or she must perform, which are regulated by operational prescriptions in the workplace. Among the figures involved in this change and expansion of the job description, we find the figure of the physiotherapist. Regarding the figure of the physiotherapist, since the COVID-19 pandemic, it is preferred that when we mention a therapist with extended tasks toward digital in person or remotely (for example in remote therapy), we refer to the *augmented physiotherapist* (APT) or *digital physiotherapist* (DPT). This figure must be rethought starting from the new interaction tasks emerging in the COVID-19 era with the looming social distancing. Furthermore, the physical and rehabilitative medicine sector is moving in this direction. For some years now, there has been talk of new forms of therapy delivery in this area in virtual mode through remote digital communication or using new tools such as the SRs. For example, Alam Le has focused on this and analyzed the critical issues highlighted in the current pandemic and the previous pandemic experiences, analyzed the changes already requested by some key figures of the health system in relation to technologies due to new intervention models consolidated during the current epidemic, and reported some consensus studies on digital rehabilitation focused around the new figure of the *DPT* without forgetting the ethical and curricular aspects [5].

The SRs in their collaborative interaction have many capabilities: establishing and maintaining social relationships; learning social skills development and role models; using “natural” signals, such as gestures and gaze; expressing emotions as well as perceiving them; communicating with high-level dialogue; and expressing one’s own personality and distinctive character. We can use SRs for a variety of purposes; for example, as educational tools and therapeutic aids [2,3]. There are several examples of SRs designed for use by elderly people [6,7,8,9] but also for frail and/or handicapped subjects needing rehabilitation and assistance: for example [10,11,12,13,14,15], to support certain motor activities; support during feeding; support during displacements; support them in drug therapy—for example, by reminding them to take a drug; support them from a cognitive point of view—for example, by stimulating them with games and supporting them from the point of view of communicative interaction, even as simple company; provide support as a hospital assistant; provide support as a mediator to therapists and/or relatives.

Furthermore, in the COVID-19 era, there has been an increase in the use of SRs in the above-listed desirable activities due to the necessary supervening obligation of social distancing to combat the pandemic [2]. The COVID-19 pandemic has created an unprecedented incentive for the development of the technologies in healthcare. This development involved both the boost and regulation of already consolidated solutions and the exploration of new potentials. All this certainly concerned digital health in the countless applications of *mHealth* and *eHealth*, but it also concerned other technologies, such as mechatronics applied in healthcare as in rehabilitation and assistance robotics [16]. Among the mechatronic devices that have had an important push in this period, we certainly find the SRs. If we focus on PubMed, we can immediately see how in 2020 (the year of the pandemic), we had 413 publications on SR, which was an increase of 24.8% compared to the number in 2019. In the first 4 months of 2021, furthermore, we already have 190 publications on SR, which is a trend that if confirmed by the end of the year could lead us to almost double the number of publications compared to those of 2019. As already highlighted for *mHealth* and *eHealth* technologies, it is particularly important to analyze the impact of innovative technologies on humans at work and in living environments. Regarding artificial intelligence, in a previous study, we analyzed the importance of the consent of digital radiology operators in view of a post-pandemic use through the proposition of targeted/calibrated surveys for those who will then have to work with technology [17]. In social robotics, the focus of this study, powerful efforts in algorithms are being made through artificial intelligence to allow continuous improvement of the SR in carrying out its role as an interaction with the human subject. A lot is expected of artificial intelligence (AI) in these devices. We expect the elimination or minimization of weaknesses of the mechatronic system such as the lack of empathy, of psychological perception, and of the capacity for discernment, which are all fundamental aspects if you want to position this device firmly in the role of collaborator and/or professional assistant. The AI is currently used to face this [18,19,20,21,22,23,24,25]: for example, to (a) help in recognizing facial expressions [18,19] and consequently propose adaptations; (b) improve aspects such as empathy [24,25]; (c) adapt the environments of life built around the individual [23]; (d) improve the acceptance and the prospects for the use of these technologies [20,21,22]. As in the studies proposed in [26,27], here too, we feel the need to investigate the consensus of the figures involved (physiotherapists) on the use of innovative AI-based devices that can radically change work patterns. Many figures are revolving around the SRs, ranging from the bioengineer to the physiotherapist without neglecting the stakeholders. One of the key figures regarding the interaction with the SR is that of the physiotherapist; therefore, the consensus of this figure around social robotics is strategic.

The purpose of the study was to investigate the consensus of the physiotherapists around the introduction of the SRs in the clinical practice both in rehabilitation and assistance. To achieve the main goal, we have decided to (a) develop a tool based on an *electronic survey* (eS) focused on the social robotics applied to the rehabilitation and assistance; and (b) submit the eS to physiotherapists to investigate their opinion and their level of consent on the topic. We have also decided to assess the acceptance of the eS on the physiotherapists who have participated in the study, in consideration of future uses in this area. An *electronic feedback form* (eFF) was designed for this.

The study is organized as follows.

*Section Two* (I) describes the methodology used in the technological choice, development, and administration of the electronic survey; it also (II) reports the inclusion protocol and study participants. *Section Three* reports (III) the output relating to the administration of the survey to the participants included in the study, divided by the types of application forms used (graded questions, Likert, and multiple choice), and (IV) the respondents’ feedback on the electronic survey and employment prospects. *Section Four* discusses the evidence that emerged from the study and in particular, (V) the degree of consensus/acceptance on the introduction of the SR in healthcare and (VI) the high acceptance of the method, based on an electronic survey, as a periodic monitoring tool.

## 2. Materials and Methods

We have decided to develop the survey electronically; this allows both ease of administration using very convenient IT tools and ease of data collection. These tools have also the possibility of automatic reporting. Microsoft Forms was chosen in this study. It is available in the Microsoft 365 App Business Premium suite provided in the workplace. All users can access through their own domain account guaranteed by the corporate cybersecurity standards (which must comply with the international regulations in force) supported both by the *system security tools*/*system policy* and network *security*. The use of both an internal recommended tool (respecting the cybersecurity) and the plan to submit the *eS* anonymously simplified the authorization process (see the footnote).

We developed the sections of the eS with different types of questions: open question, choice, multiple choice, Likert, and graded questions. In the graded questions and the Likert, we fixed a six-level psychometric scale; therefore, it was possible to assign a minimum score of one and a maximum of six with a theoretical mean value (TMV) of 3.5. We can refer to the TMV for comparison in the analysis of the answers. An average value of the answers below TMV indicates *a more negative than positive response*. An average value above TMV indicates a *more positive than negative response*. In consideration of the objective of this study and the survey, we also managed the survey as a virtual focus group with careful considerations to the consensus issues related to all the aspects of the introduction of the SR. We started from the training up to the relationships between the SR in the several potential activities of involvement, with also the idea to create a stable product for the scientific societies. The study was designed at the Catholic University (CU) headquarters in Rome and San Martino al Cimino (Viterbo) and ran from 15 May 2021 to 15 July 2021. Regarding the address of the survey, we turned to physiotherapists under their course of the study (*PUCS*) and after the course of the study (*PACS*). We considered new graduates from less than a year to belong to the first group and those who then undertook a further specialization to belong to the second. The minimum age was 23 years; the maximum age was 58; Table 1 reports the demographic data.

Therefore, we disseminated it with respect to the current regulations (see footnote at the end) using the mobile technology through social media, such as Facebook, LinkedIn, Twitter, Instagram, and WhatsApp; scientific and professional associations; and, in general, a *peer-to-peer* dissemination to collect data in the extended territory and therefore not limited to the CU.

We also submitted to all participants an electronic feedback form (*eFF*) based on the same technologies with a few questions of a graded evaluation type to investigate the acceptance of the eS in term of the robustness of the tool and to investigate the prospects.

We planned a dedicated post-processing analysis of the eS after submission.

The WEB link of the interactive tool eS is reported in [28]. The printout of the eS is reported in [29].

## 3. Results

The results are organized in two parts:

- Output of the survey administration.
- Output related to feedback on acceptance of the proposed electronic survey methodology and prospects.

### 3.1. The Outcome of the Electronic Survey

*The questionnaire in brief*

The questionnaire includes *25 questions*, as anticipated, of different types, including open questions, to have as broad a view as possible. *Questions 1–7* collect information about the informant (age, sex, training received, and membership in scientific societies). The *graded questions 8*, *12–13*, and *19–20* are on the knowledge of SRs in general, on the impact in the world of assistance and physiotherapy, and on the influence of AI and ethics. *The questions through Likert modules 10, 14, and 15* address the detailed knowledge and the strengths and weaknesses perceived on the SRs. The *multiple-choice questions 16–18* address aspects related to the *workflow* changes and the relative role of the SR and physiotherapist. The remaining *questions 9*, *21–25* accompany the others and/or are designed to indicate further wishes in this area.

*First considerations*

The age distribution of the two samples was normal, both when they were considered separately and jointly. Since the number of recruits exceeded 50 for each sample, the Kolmogorov–Smirnov test was chosen and preferred to Shapiro–Wilk [30,31,32]. To the question relating to knowledge in general, “*Q8. What is your level of knowledge of social robots in general?”*, the PUCS group reported an average score of 3.83, while the PACS group reported an average score of 3.87. Student’s *t*-test showed no significant differences (*p* = 0.009) [33].

*The first analysis on the graded questions*

The results in Table 2 show, for all the recruited, an average value above TMV (3.5) that indicates a more positive than negative response. Results show a high value in the answers to the questions:

- *Q12. How useful do you think the social robot can be in physiotherapy?*
- *Q13. How useful do you think the social robot can be in assistance?*

In addition, the answer to “*Q19. Do you think that the artificial intelligence will help improve this device by eliminating weaknesses?*” showed great confidence in artificial intelligence.

The participants consider ethics as an obstacle to the spread of the device, as evidenced by the high score given to the answer “*Q20. Do you think that issues relating to ethics will be an obstacle to the spread of this device?*

Table 2 shows that the percentage was never lower than 82.05%, which gave a value ≥ 4, with a high significance (*p* < 0.01, test χ2). For the two groups considered separately, the χ2 test reported the same significance (*p* < 0.01).

*The second analysis on the Likert questions*

The average values, for all the recruited, were above TMV (3.5) for all the Likert questions. This indicates a more positive than negative response for all three Likert questions:

- *Q10. Degree of knowledge on social robots?*
- *Q14. What are the strengths of social robots?*
- *Q15. What are the weaknesses of social robots?*

Table 3 reports the most two popular questions for each Likert.

For Likert *Q10*, we had “*Robots for the elderly*” and “*Robots for people with communication disabilities*”.

For Likert *Q14*, we had “*It does not judge*” and “*reliable*”.

For Likert *Q15*, we had “*Lack of empathy*” and “*Risk of false relationships*”.

Table 3 shows that the percentage was never lower than 86.69%, which gave a value ≥ 4, with a high significance (*p* < 0.01, test χ2). For the two groups considered separately, the χ2 test reported the same significance (*p* < 0.01).

*The third analysis on the multiple choice questions*

We report also for the multiple choice questions the two most popular answers.

Table 4 shows the outcome.

For question Q9, “Where did you hear about it?”, the most two popular statements were “Internet” (number of votes = 175) and “University” (number of votes = 168). The respondents also had the possibility of indicating “Other” among the answers. This possibility eventually allowed those who had had direct knowledge of the SRs in the field to make it explicit and detailed. No one has selected this field to indicate direct acquaintance. This is in line with the national situation where the use of these systems is still rare.

For question Q16, “I think in the future, the social robot…”, the most two popular statements were “It will be useful but complementary” (number of votes = 194) and “It will not catch on” (number of votes = 147).

Questions Q17 and Q18 are particularly strategic in consideration of the impact on the model of work in the field and on the revisiting of the job description (changing with the modifications of the workflow) of the future DPT and/or APT.

For question Q17, As a physiotherapist, how can I be useful to the social robot?, the most two popular statements were “As an operational manager of its use” (number of votes = 168) and “In performance monitoring” (number of votes = 155).

For question Q18, How will the social robot be useful to my profession?, the most two popular statements were “Increase in working capacity” (number of votes = 158) and “Facilitates integration with other professionals” (number of votes = 147).

### 3.2. The outcome of the Electronic Feedback Form

An important aspect of our survey is that relating to the opinion on the usefulness of the proposed questionnaire. A total of 312 out of 323 participants submitted feedback. Table 4 shows, on a six-value scale, the high acceptance of the methodology both in terms of the prospects of the survey in general (Questions 1–3) and some important characteristics taken into consideration (Questions 4–8). The table shows the following:

- All average ratings are above 5.0;
- No minimum rating is less than 4 (>TMV = 3.5), indicating that in all cases and for all questions, the instrument has always received a positive rating (more yes than no);
- The question “Evaluate the survey in general as a specific tool for the social robot” received the highest score, clearly indicating an important perspective for using the survey tool.

## 4. Discussion

The COVID-19 pandemic has created an unprecedented incentive for the development of the technologies in healthcare. This development involved both the boost and regulation of already consolidated solutions and the exploration of new potentials.

All this certainly concerned digital health in the countless applications of *mHealth* and *eHealth* but also other technologies, such as mechatronics applied in healthcare as in rehabilitation and assistance robotics [16]. Among the mechatronic devices that have had an important push in this period, we certainly find the SRs [1]. Many professional figures are revolving around the SR device, ranging from the bioengineer to the physiotherapist without neglecting the stakeholders (who in a rationalization of resources can also be economists). Among these figures, we find that for the physiotherapist, we are witnessing two phenomena. The first is that the physiotherapist is increasingly becoming a figure that has to do with *Digital Health*, so much so that today, we talk about an *augmented physiotherapist* (APT) and/or *digital physiotherapist* (DPT) [5]. On the other hand, the SRs through research are improving more and more in the aspects of social interaction thanks also to artificial intelligence [18,19,20,21,22,23,24,25].

It begins to become important or even strategic to investigate how SRs and physiotherapists are approaching and becoming familiar.

In this study, we have proposed a useful investigation, in view of consensus studies/conferences/guideline that can be used for the introduction of methods based on SRs in rehabilitation practices.

Therefore, we have developed an electronic survey focused on social robot-based rehabilitation and assistance and submitted it to physiotherapists in the field or in training to investigate their opinion and their level of consent regarding the use of the SR in rehabilitation and assistance. The outcome of the study has several polarities.

A *first polarity* consists of having designed a methodology based on the electronic surveys that allows the investigation of different aspects of the introduction of the SRs and on the relevant relationship with the figure of the DPT (or APT).

The *second polarity* consists on having verified by the physiotherapists the consensus/acceptance on the introduction of the SR in the healthcare. From the analysis of the subjects involved in the study, the following emerged in particular:

1. A coherent consensus and acceptance;
2. A high degree of knowledge of these systems;
3. The clear conviction on: (a) the usefulness of these systems in both rehabilitation and assistance; (b) that the artificial intelligence will be of aid in reducing the weakness of the device and (c) the ethical issues will hamper the use of this device;
4. A coherent vision on the strengths and the weakness of this device as highlighted in the Likert questions. In particular, among the strengths, the most voted were “It does not judge” and “It is reliable”; while among the weakness, the most voted were “The lack of empathy” and “The risk of false relationships”.

The *third polarity* consists of having investigated, using multiple-choice questions, the vision strictly related to the evolution of their *job description* correlated to the changes of the *workflow* with the introduction of the SR. The physiotherapists are convinced that (a) they will be particularly useful with the SR both “*As an operational manager of its use*” and “*In performance monitoring*; (b) the SR will particularly aid them in the “*Increase in working capacity*” and “*Facilitating the integration with other professionals*”.

The *fourth polarity* consists of an acceptance of feedback from the figures involved, in relation to the electronic tools of investigation proposed in the study. This feedback is useful for planning future initiatives and interventions.

From a general point of view, the study presents four added values.

The *first added value* is the product [28,29] represented by the electronic survey tool that can be easily submitted through the *mobile technologies* on the net during the pandemic. The *second added value* is represented by the survey with a wide range of aspects related to the use of the SRs and the direct impact on the *workflow* and therefore on the *job description* of the physiotherapist, having more and more to interact with the digital technologies in the pandemic era. The *third added value* is represented by the possibility of using this product, after minimal changes even in non-pandemic/post-pandemic periods for example by scientific and/or professional societies, to monitor as both a technological and social sensor the evolution of the topic. The *fourth added value* is represented by the outcome with reference to the two groups of *PUCS* and *PACS*, which is promptly useful for the stakeholders. The *fifth added value* lies in the outcome of the feedback form, which highlights how the tool has been appreciated both in terms of design and effectiveness of administration and how it is believed that it can be useful in the hands of scientific societies for periodic monitoring.

An important message emerges from the study for stakeholders. They must consider that the technological evolutions of SRs represent an unstoppable process, as well as the increase in the aging of the population [26,27]. They must not look with suspicion toward these devices, which can represent an important resource, but rather invest in monitoring and consensus training initiatives also through survey tools [17].This study certainly has the limitation of not having been able to administer a sweeping survey and on all professional figures, but it has the advantage of having proposed a useful, accepted automatic tool and the application of the survey methodology on a first sample that shows important evidence. From a general point of view, this article supports the initiatives that aim to facilitate the work of the physiotherapists when using the SRs with clear rules and a highly shared consensus. Future developments of the study foresee, after further targeted data-mining, an improvement of the electronic survey and a standardization of the same as a tool in the hands of scientific societies for periodic monitoring and investigations useful for making decisions and making improvements in the introduction of technology into the work routine.

## 5. Conclusions

SRs are bursting into health systems and playing a key role in many sectors, including rehabilitation [2]. The recent pandemic has accelerated this process [1]. It is foreseeable that in the coming years, many professionals in the health sector will have to deal with these devices through new working models based on SRs [26,27]. These systems involve and will involve figures who have to do with the elderly [6,7,8,9], frail, and handicapped individuals with motor and communication problems [10,11,12,13,14,15]. These systems involve and will involve figures who have to do with the elderly, frail, and handicapped individuals with motor and communication problems. Physiotherapists are certainly among the key figures, and recently, and in the pandemic period, they have had to deal more and more with digitization processes [5]. In this study, we focused on the figure of the physiotherapist, and we prepared a survey focused on the consensus and opinion of the use of this device. This study involved submitting an electronic survey on two statistically independent samples to collect and analyze the data automatically. The survey showed a consistency of the results on the investigated sample from which interesting considerations emerge.

Contrary to stereotypes that report how AI-based devices put jobs at risk; physiotherapists are not afraid of these devices. Physiotherapists believe that SRs can be reliable co-workers who do not judge. They believe that yes, SRs have weaknesses such as the lack of empathy and they risk creating false relationships, but they also believe that artificial intelligence on the one hand and wise professional use on the other will help overcome these limits. Physiotherapists also believe that SRs will remain a complementary tool and that their role will be of the utmost importance as an operational manager of its use and in performance monitoring. These professionals also believe that the device will allow an increase in working capacity and facilitate integration with other professionals.

All those involved in the study believe that the proposed electronic survey has proved to be a useful and effective tool that allows an *instantaneous creation of virtual focus groups*. They believe in this tool and believe that it can be useful as a periodic monitoring tool and useful for scientific societies.

## Figures and Tables

**Table 1:** Characteristics of the participants in the two electronic-based submissions: the PUCS and the PACS.

Submission	Number Invited	Participants	Males/Females	Min Age/Max Age	Mean Age
*Physiotherapists under the course of the study* *(PUCS)*	161	156	79/77	23/35	24.3
*Physiotherapists after the course of the study* *(PACS)*	170	167	86/81	25/58	42.4

**Table 2:** Results relating to the graded questions with the details of the assessment.

Question	N(1)	N(2)	N(3)	N(4)	N(5)	N(6)	Mean
*Q12.How useful do you think the social robot can be in physiotherapy?*	9	17	8	98	102	89	4.65
*Q13. How useful do you think the social robot can be in assistance?*	10	8	2	52	115	136	5.04
*Q19. Do you think that the artificial intelligence will help improve this device by eliminating weaknesses?*	1	4	11	3	129	175	5.41
*Q20. Do you think that issues relating to ethics will be an obstacle to the spread of this device?*	9	1	8	7	87	211	5.46

**Table 3:** Results related to the two most popular answers of each Likert with the details of the assessment.

Likert/-Most Popular Answer-	N(1)	N(2)	N(3)	N(4)	N(5)	N(6)	Mean
*Q.10. Degree of knowledge on social robots?/-Robots for the elderly-*	12	9	14	27	85	176	5.14
*Q.10. Degree of knowledge on social robots?/-Robots for people with communication disabilities-*	14	13	16	28	84	168	5.04
*Q.14. What are the strengths of social robots?/-It does not judge-*	4	10	5	12	90	202	5.41
*Q.14. What are the strengths of social robots?/-Reliable-*	10	11	10	96	39	157	4.90
*Q.15. What are the weaknesses of social robots?/-Lack of empathy-*	9	12	7	27	87	181	5.21
*Q.15. What are the weaknesses of social robots?/-Risk of false relationships-*	7	12	10	97	37	160	4.93

**Table 4:** Feedback form output.

Question	N(1)	N(2)	N(3)	N(4)	N(5)	N(6)	Mean
Evaluate the survey as a tool for periodic monitoring and useful for the scientific societies	0	0	0	96	79	137	5.13
Evaluate the survey as a tool to obtain structured information from virtual focus groups	0	0	0	99	77	136	5.11
Evaluate the survey in general as a specific tool for the social robot	0	0	0	58	93	161	5.33
How user-friendly was the tool?	0	0	0	94	80	138	5.14
How effective was the tool	0	0	0	111	81	120	5.02
How complete was the tool?	0	0	0	74	89	149	5.24
How clear was the tool?	0	0	0	104	88	120	5.05
How functional was the tool?	0	0	0	103	74	135	5.10

## Data Availability

Not applicable.
